# Correction to “The Role of Platelet‐Rich Plasma in Androgenetic Alopecia: A Systematic Review”

**DOI:** 10.1111/jocd.70882

**Published:** 2026-06-18

Donnelly C, Minty I, Dsouza A, et al. The Role of Platelet‐Rich Plasma in Androgenetic Alopecia: A Systematic Review. *J Cosmet Dermatol*. 2024;23:1551–1559. 10.1111/jocd.16185.

In the Affiliations section, a second affiliation was incorrectly attributed to author A. Khajuria. This affiliation has been removed, and the author’s affiliation has been corrected accordingly.

The online version of this article has been updated accordingly.

We apologize for this error.

